# Supplementary material for: Institutions and the resource curse: New insights from causal machine learning
Source: PLoS One. 2023 Jun 1;18(6):e0284968. doi: 10.1371/journal.pone.0284968 (PMC10234542; doi:10.1371/journal.pone.0284968)
Supplement: S1 Appendix — (DOCX) [file pone.0284968.s001.docx]

# Online Appendix

## A.1: Data sources for world market prices of minerals

Table A1: Commodity Shorthand, Name, Data, and Price Source

| **Abb.** | **Name** | **Financial Market Data** | **Source** |
| --- | --- | --- | --- |
|  |  |  |  |
| Ag | Silver | Silver World Prices | World Bank |
| Au | Gold | Gold Bullion LBM | SNL - Thomas Reuters |
| Bx | Bauxite | Bauxite World prices | USGS Commodity Prices |
| Ch | Chromite | Chromium 99%min FOB China | USGS Commodity Prices |
| Co | Cobalt | Cobalt World Prices | USGS Commodity Prices |
| Coal | Coal | South African Index, Coal Report | World Bank |
| Cu | Copper | LME-Copper Grade A Cash | SNL - Thomas Reuters |
| Diam | Diamonds | Industrial Grade Diamonds | USGS Commodity Prices |
| Gph | Graphite | Natural Graphite Prices | USGS Commodity Prices |
| Ilm | Ilmenite | Iron Oxide Pigment Prices | USGS Commodity Prices |
| Fe | Iron | Iron Ore 62% China Imp CFR | World Bank |
| Lanth | Lanthanides | Rare Earth Elements World Prices | USGS Commodity Prices |
| Li | Lithium | Lithium World Price | USGS Commodity Prices |
| Mg | Manganese | Manganese World Prices | USGS Commodity Prices |
| Nd | Niobium | Niobium Pentoxide 99.5% FOB China | USGS Commodity Prices |
| Ni | Nickel | LME-Nickel Cash | World Bank |
| U3O8 | Triuranium  Octoxide | Uranium U308 Restricted Price  Nuexco Exchange | International Monetary Fund |
| Pb | Lead | Lead, 99.97% pure, LME Cash | World Bank |
| Pd | Palladium | LME - Palladium | SNL - Thomas Reuters |
| Ph | Phosphate | Phosphate Rock, Morocco, 70% BPL | World Bank |
| Pot | Potash | Potassium Chloride Standard Grade, Vancouver | World Bank |
| Pt | Platinum | UK 99.9% Refined, London Afternoon Fixing | SNL - Thomas Reuters |
| Rut | Rutile | Titanium Dioxide Pigment Prices | USGS Commodity Prices |
| Sn | Tin | LME-Tin 99.85% Cash | SNL - Thomas Reuters |
| Sv | Antimony | Antimony 99.65% CIF NEW | USGS Commodity Prices |
| Ta | Tantalum | Tantalum Pentoxide World Prices | USGS Commodity Prices |
| V | Vanadium | Vanadium Pentoxide min 98% | USGS Commodity Prices |
| W | Tungsten | Tungsten Oxide WO3 99.95% FOB | USGS Commodity Prices |
| Y | Yttrium | Y Oxide 99.999%min China | SNL - Thomas Reuters |
| Zn | Zinc | LME-SHG Zinc 99.995% Cash | SNL - Thomas Reuters |
| Zr | Zircon | Zirconium World Prices | USGS Commodity Prices |

*Sources:*

International Monetary Fund (IMF): http://www.imf.org/external/np/res/commod/index.aspx

SNL: http://www.snl.com/Sectors/metalsmining/Default.aspx

United States Geological Survey (USGS): http://minerals.usgs.gov/minerals/pubs/mcs/

World Bank: http://data.worldbank.org/data-catalog/commodity-price-data

All pages accessed in July 2016.

## A.2 Definition and sources of confounding variables

This appendix describes the confounding district- and country-level variables used in our analysis in more detail. It also describes the data sources and therefore complements the information in Section 3.5.

### *District-level variables*

The district-level variables are mostly based on geo-spatial data and computed using the ADM2 boundaries from the GADM database of Global Administrative Areas (Version 1).

*Area:* Land area computed based on GADM shapefiles of administrative boundaries.

*Distance to Capital:* The log of the distance between the district’s geographic center and the country’s capital in km. The district’s centroid was calculated by the authors. Information about the coordinates of a country’s capital is taken from Weidmann et al. (2010).

*Distance to the Coast:* The log of the distance between the district’s geographic center and the nearest coastline in km. The district’s centroid was calculated by the authors. Vector data on the world’s shorelines stems from Wessel and Smith (1996).

*Elevation:* We use data from GTOPO30, which is a global digital elevation model (DEM) with a horizontal grid spacing of 30 arc seconds (approximately 1 km), to calculate each district’s minimum/median/maximum elevation. These data are distributed by the USGS EROS Archive.

*Land use (11 share variables):* We use data from the Global Land Cover Characterization (GLCC) database, which contains raster data files with a resolution of approximately 1km that classifies the land cover of an area over the period 1992 to 1993. We calculate for each district the share of the land area with the following 11 types of land cover: artificial, crop, grass, trees, shrubs, herbaceous, mangroves, sparse, bare soil, snow, and water. This database is distributed by the USGS EROS Archive.

*Land suitability for agriculture:* Average land suitability for agriculture within each district. The index calculates land suitability for cultivation based on climate and soil constraints. This variable is missing for some observations, as the original raster data does not provide complete coverage of the globe’s land area (e.g., it does not cover some peninsulas and islands). The raw raster data comes from Ramankutty et al. (2002).

*Number of traditional ethnic homelands:* We use data by Murdock (1959, 1967) who mapped the spatial distribution of over 800 African ethnicities around colonization. We use the shapefiles mapping the ethnic homeland boundaries and overlay them with the district boundaries to calculate the number of traditional homelands that intersect the districts.

*Ruggedness:* We use data from GTOPO30 to calculate the Mean Terrain Roughness Index. This index reflects the average absolute height difference between a raster pixel and its neighbors and is normalized to 0–1. These data are distributed by the USGS EROS Archive.

*Pre-colonial centralization:* We use data by Murdock (1959, 1967) who mapped the spatial distribution of over 800 African ethnicities around colonization and compiled information on their pre-colonial political centralization, among others. We assign each district the pre-colonial political centralization corresponding to the ethnic homeland in which it is located. For districts that intersect more than one ethnic homeland, we assign the pre-colonial political centralization of ethnic homeland that covers the largest part of the district.

*Population and Population Density:* The log of the number of people and people per km^2^ in the district. Population data stems from the Gridded Population of the World, Version 3 (CIESIN, 2016).

*Ports (with and without oil terminals):* We use point locations of major ports and oil terminals in Africa from the World Port Index by the National Geospatial-Intelligence Agency and classify a district as a port (a port-with-oil-terminal) district if one or more ports (with an oil terminal) are located in that district.

*Temperature and precipitation:* The data on temperature and precipitation stems from the 1900–2014 Gridded Monthly Time Series, Version 4.01 (Willmott and Matsuura, 2015) that contains monthly and annual average air temperature and total precipitation based on ground station data measurements. The raw data are GIS raster files with a cell size of 0.5 × 0.5 degrees (approx. 56 km × 56 km at the equator) which we combine with shapefiles of the ADM2 boundaries and calculate zonal means for each region and year.

### *Country-level variables*

The second set of confounding variables vary at the country and year level. This set contains countries; years; country-years; indicators for the former colonial rulers; the indices of ethnic and religious fractionalization by Alesina et al. (2000); GDP per capita (in constant 2010 USD and as annual growth rate) and inflation from the World Development Indicators; and a large set of institutional variables. The institutional variables include: the indices of Civil Liberties and Political Rights by Freedom House; the Polity2 score by the Polity IV Project; the six Worldwide Government Indicators (i.e., Control of Corruption, Government Effectiveness, Political Stability, Regulatory Quality, Rule of Law, Voice and Accountability) by the World Bank; as well as information on whether the political system is presidential or parliamentary by the World Bank’s Database of Political Institutions; and whether it is unitary or federal by the Institutions and Elections Project. These institutional variables and their sources are all described in more detail in Teorell et al. (2023).

## A.3. References

Alesina, A., A. Devleeschauwer, W. Easterly, S. Kurlat, and R. Wacziarg (2003): Fractionalization, *Journal of Economic Growth*, 8(2), 155–194.

Center for International Earth Science Information Network (CIESIN, 2005): *Gridded Population of the World, Version 3 (GPWv3): Population Count* (Palisades, NY: NASA Socioeconomic Data and Applications Center – SEDAC).

Murdock, G.P., 1967: *Ethnographic Atlas* (Pittsburgh: University of Pittsburgh Press, 1967).

Murdock, G.P., 1959: *Africa: Its Peoples and Their Culture History* (New York: McGraw-Hill).

Ramankutty, N., J.A. Foley, J. Norman, and K. McSweeney (2002): The Global Distribution of Cultivable Lands: Current Patterns and Sensitivity to Possible Climate Change, *Global Ecology and Biogeography,* 11(5), 377–392.

Teorell, J., A. Sundström, S. Holmberg, B. Rothstein, N. A. Pachon, C. M. Dalli, and Y. Meijers (2023): *The Quality of Government Standard Dataset, version Jan23*, University of Gothenburg.

Weidmann, N.B., D. Kuse, and K.S. Gleditsch (2010): The Geography of the International System: The CShapes Dataset, *International Interactions*, 36(1), 86–106.

Wessel, P., and W.H.F. Smith (1996): A Global Self-consistent, Hierarchical, High-resolution Shoreline Database, *Journal of Geophysical Research: Solid Earth*, 101(B4), 8741–8743.

Willmott, C. J. and K. Matsuura (2015): Terrestrial Air Temperature and Precipitation: Monthly and Annual Time Series (1900–2014), University of Delaware.
