# Supplementary material for: Institutions and the resource curse: New insights from causal machine learning
Source: PLoS One. 2023 Jun 1;18(6):e0284968. doi: 10.1371/journal.pone.0284968 (PMC10234542; doi:10.1371/journal.pone.0284968)
Supplement: Attachment — Submitted filename: Report on PONE-D-22-33969.pdf [file pone.0284968.s002.pdf]

Report on PONE-D-22-33969

Institutions and the Resource Curse: New Insights from Causal Machine Learning

By Roland Hodler, Michael Lechner, and Paul Raschky

Summary: The paper "*Institutions and the Resource Curse: New Insights from Causal Machine Learning*" empirically examines whether institutions impact the relationship between natural resources and economic development and political violence. The authors use data for 3,800 ADM2 regions in Sub-Saharan Africa observed over the years 1996-2013 and employ causal forest estimators to re-evaluate the Mehlum et al. (2006) hypothesis that natural resources impact growth and violence differently depending on the strength of institutions. The results are broadly in line with those presented in previous studies on the topic (i.e. stronger institutions increase the positive effect of mining activities on economic development and decrease the potential of mining-induced violence).

Overall assessment: The paper addresses an important research question that has been examined by a battery of studies during the past years and decades. The main innovation of the paper is to employ causal forest estimators to study whether the resource-development nexus depends on the strength of institutions. For this particular setting, I agree with the authors that the causal forest estimator provides some valuable statistical properties. One of the authors is actively involved in developing this approach further, and so quite unsurprisingly, the authors demonstrate excellent command of this technique and use the approach very convincingly. I have no doubt that the results are credible, but the findings are also very much in line with the results of previous studies on the topic. Hence, the contribution to the literature, in this respect, is limited. It was also a bit unclear to me in how far the paper aims to provide a technical contribution (in which case the description of the main method compared to other approaches should be strengthened) or mainly a content-specific contribution (in which case the results should be discussed in much greater detail against the backdrop of previous findings). In general, I think that the methodology employed in the paper can be very valuable also in other settings, and I think that the paper *does* find results (particularly non-linearities) that we would not typically expect when using traditional methods. These differences should however be discussed in a more systematic way. I also have some additional comments on the paper and the empirical method that I discuss in detail below.

COMMENTS

- **The contribution to the literature**: I agree with the authors that the employed causal forest estimator has favorable statistical features in the employed stetting. A particular advantage is to look at group specific treatment effects (GATEs), which allow to study

the effect of natural resources on development and violence depending on the strength of institutions. However, the results are broadly in line with those of previous studies. So essentially, what the paper does is to provide support for the findings of previous work using an approach that is, as the authors put it, "*preferable or, at least, a worthwhile alternative*" (Page 3). In this respect, I think that even though the analysis is interesting, the contribution to the literature is limited. In order to convincingly argue that the approach is valuable (and more valuable than previous methods), I think the authors can do more to demonstrate that their approach delivers results that differ from those that we would typically be able to detect using traditional approaches. The non-linearities identified in the analysis provide a promising avenue for such a discussion. I also think the paper could make a more general point in discussing the (undoubtedly large) potential of the utilized method for research in (developing) economics in a more general manner

- **The motivation underlying the use of the causal forest estimator:** Related to my previous comment, the authors motivate the use of their approach by highlighting some problems of traditional methods typically used to study topics such as those in the manuscript, referring to a burgeoning strand of the econometric literature that emphasizes that heterogeneity in the treatment effects can bias the results of difference-in-differences estimations and event study designs. The authors mainly refer to de Chaisemartin and D'Haultfœuille (2020), but there are many other recent papers that address similar topics (e.g. Sun and Abrahams, 2021, "*Estimating dynamic treatment effects in event studies with heterogeneous treatment effects*", Journal of Econometrics; Borusyak et al., 2022, "*Revisiting Event Study Designs: Robust and Efficient Estimation*"). These papers *do* provide methodological frameworks to precisely account for the concerns mentioned by the authors, so motivating the authors' empirical method by the concerns mentioned by de Chaisemartin and D'Haultfœuille (2020) does not help much. Regarding the second argument, I share the authors' critique of the standard linearity assumption, but then I wonder whether the authors' findings (i.e. finding the same results as previous studies) alleviate concerns about a bias initiated by a "wrong" functional form? Related to my first comment, the non-linearities found in the analysis should be emphasized more and discussed against the backdrop of the findings of previous studies. More generally, I think that the motivation for the use of the causal forest estimator should be strengthened.

- **The empirical strategy**: The authors demonstrate great mastering of the causal forest estimator and employ this approach in a convincing way. Nevertheless, I think that there are some open questions (of general nature and, in particular, for readers not familiar with this type of approach) that should be addressed:

    - The conditional independence assumption (referred by the authors as "CIA") requires that no variables other than those included in their model influence the treatment and the potential outcomes. For analyses that study macroeconomic effects (even though the units of observation are ADM2 regions), this assumption is most likely never fulfilled. The authors argue that it is plausible

to assume that the CIA is fulfilled in their setting, because they add a large number of covariates. While I think that the probability for selection on unobserved factors may potentially be low in the authors' setting, this point requires more discussion. The paper motivates the study by citing the "credibility revolution" in economics and criticizes prior methods that aim to tackle unobserved omitted factors in section 2. I think that, particularly when making such strong statements, it is unsatisfactory to say that the problem can be solved by simply adding more variables (although in a quite different methodological setting, this was the core idea underlying previous methodological innovations, e.g. Extreme Bound Analyses in the 1980s/1990s and LASSO regressions in the 2010s). More generally, I think that the empirical analysis would be more convincing if the authors included a brief discussion on how they expect unobserved variables to influence their results.

- Another important assumption of the analysis is that the availability of resources is exogenous. On Page 8, the authors write that "*along with the recent subnational literature, we argue that the availability of natural resources at the district level is mainly shaped by geology and, therefore, exogenous*". Can we really say that this is the case? What about political factors or effort invested to find natural resources? Even geology itself may be endogenous, i.e. when factors rooted in years before 1996 (the starting point of the author's sample) have changed the geological circumstances and facilitated access to natural resources? These factors may well be corelated with growth (via budget constraints) and conflict (by decreasing the potential to be productive in searching for natural resources). But if availability of natural resources would be truly exogenous, then untreated units would never have the chance to be treated at all. If this is case, then I wonder whether they can serve as a plausible control group, i.e. we cannot produce a counterfactual of the treated units using observable characteristics, because the characteristics of treated and untreated units are different "by nature". This problem is not specific to the authors' setting and there is some discussion on this point on Page 9. This discussion, however, is a bit vague and it is not clear to me how severely the "*substantial overlap problems*" the authors find affect the empirical results.
- When describing their method, the authors acknowledge that their strategy comes at "*the cost of not being able to include that many fixed effects*" (Page 6). To account for heterogeneity across units and years, the authors include some "indicator variables", but from their very brief description, it was not clear to me what these variables measure. If the model includes indicator variables for country-years, then how can additional variables that only vary at the country-year level (such as country-level ethnic diversity) add any additional information? The answer may potentially lie in the nature of the indicator variable, but this should be explained briefly in the text.
- I missed some discussion of the numerical meaning of the results. On Page 15, for example, the authors state that "*the estimated effects imply that mining activities increase nighttime lights by 16.0 percent if mineral prices are low, and 48.3 percent if mineral prices are high*". This and other similar statements are

a bit vague, because nighttime lights are measured on a very counterintuitive scale. Also, the paper is mute about the distribution of nighttime lights. So how large, in economic terms, is an increase in nighttime lights by 16% given the sample upon which these estimates are obtained? I think it is essentially to communicate the meaning of the results in a more reader-friendly way, and the numerical meaning of the estimated effects should be provided directly in the introduction (see also my comments on the exposition of the manuscript).

- In how far are the results affected by units where nighttime lights are top-coded?

- **The exposition of the manuscript:** The paper is very well written and (in most parts) crystal clear about what it does. However, I fear that some parts of the paper are (too) lengthy and sometimes repetitive. Also, some parts of the paper read more like a book than a research paper, particularly at places in the text where the authors refer to interesting but not necessarily important side issues (mainly in the introduction and the description of the method). While I enjoyed reading the text and while there is clearly personal taste in writing, I think that the communication of the results could be strengthened by streamlining the text a bit, particularly in the introduction. Some specific examples: It takes the reader until Page 3 to learn what the paper does and until Page 4 to learn about the main results. Before, there are interesting but lengthy discussions about oil production in Guyana and the credibility revolution in economics. These are clearly interesting topics, but their relevance for the paper is minor compared to the papers' strategy and main results.
