# Supplementary material for: Institutions and the resource curse: New insights from causal machine learning
Source: PLoS One. 2023 Jun 1;18(6):e0284968. doi: 10.1371/journal.pone.0284968 (PMC10234542; doi:10.1371/journal.pone.0284968)
Supplement: Attachment — Submitted filename: Response_PLOS_R1_20230220.pdf [file pone.0284968.s003.pdf]

Reviewer #1:

The paper addresses an important research question that has been examined by a battery of studies during the past years and decades. The main innovation of the paper is to employ causal forest estimators to study whether the resource-development nexus depends on the strength of institutions. For this particular setting, I agree with the authors that the causal forest estimator provides some valuable statistical properties. One of the authors is actively involved in developing this approach further, and so quite unsurprisingly, the authors demonstrate excellent command of this technique and use the approach very convincingly. I have no doubt that the results are credible, but the findings are also very much in line with the results of previous studies on the topic. Hence, the contribution to the literature, in this respect, is limited. It was also a bit unclear to me in how far the paper aims to provide a technical contribution (in which case the description of the main method compared to other approaches should be strengthened) or mainly a content-specific contribution (in which case the results should be discussed in much greater detail against the backdrop of previous findings). In general, I think that the methodology employed in the paper can be very valuable also in other settings, and I think that the paper does find results (particularly non-linearities) that we would not typically expect when using traditional methods. These differences should however be discussed in a more systematic way. I also have some additional comments on the paper and the empirical method that I discuss in detail in the attached report.

1. In The contribution to the literature: I agree with the authors that the employed causal forest estimator has favorable statistical features in the employed stetting. A particular advantage is to look at group specific treatment effects (GATEs), which allow to study the effect of natural resources on development and violence depending on the strength of institutions. However, the results are broadly in line with those of previous studies. So essentially, what the paper does is to provide support for the findings of previous work using an approach that is, as the authors put it, "preferable or, at least, a worthwhile alternative" (Page 3). In this respect, I think that even though the analysis is interesting, the contribution to the literature is limited. In order to convincingly argue that the approach is valuable (and more valuable than previous methods), I think the authors can do more to demonstrate that their approach delivers results that differ from those that we would typically be able to detect using traditional approaches. The non-linearities identified in the analysis provide a promising avenue for such a discussion. I also think the paper could make a more general point in discussing the (undoubtedly large) potential of the utilized method for research in (developing) economics in a more general manner.

We now discuss the non-linearities as a novel insight we gain from this approach in the Introduction and the Conclusions and briefly mention it in the Abstract. We also discuss the possibility to estimate group-specific treatment effects (GATEs) as another advantage of our estimator in the Introduction and the Conclusions. We now also highlight the potential of causal machine learning estimators for research in development economics and related fields in the Conclusions.

2. The motivation underlying the use of the causal forest estimator: Related to my previous comment, the authors motivate the use of their approach by highlighting some problems of traditional methods typically used to study topics such as those in the manuscript, referring

to a burgeoning strand of the econometric literature that emphasizes that heterogeneity in the treatment effects can bias the results of difference-in-differences estimations and event study designs. The authors mainly refer to de Chaisemartin and D'Haultfœuille (2020), but there are many other recent papers that address similar topics (e.g. Sun and Abrahams, 2021, "Estimating dynamic treatment effects in event studies with heterogeneous treatment effects", Journal of Econometrics; Borusyak et al., 2022, "Revisiting Event Study Designs: Robust and Efficient Estimation"). These papers do provide methodological frameworks to precisely account for the concerns mentioned by the authors, so motivating the authors' empirical method by the concerns mentioned by de Chaisemartin and D'Haultfœuille (2020) does not help much. Regarding the second argument, I share the authors' critique of the standard linearity assumption, but then I wonder whether the authors' findings (i.e. finding the same results as previous studies) alleviate concerns about a bias initiated by a "wrong" functional form? Related to my first comment, the non-linearities found in the analysis should be emphasized more and discussed against the backdrop of the findings of previous studies. More generally, I think that the motivation for the use of the causal forest estimator should be strengthened.

At the end of section 2.1, we now reference to the difference-in-differences literature that recently developed at a rapid pace. However, we refrain from comparing our approach to the different approaches that each of these papers suggested and keep the comparison at a general level.

As stated in our response to comment 1, we now discuss the non-linearities as a novel insight we gain from this approach in the Introduction and the Conclusions and briefly mention it in the Abstract.

The empirical strategy: The authors demonstrate great mastering of the causal forest estimator and employ this approach in a convincing way. Nevertheless, I think that there are some open questions (of general nature and, in particular, for readers not familiar with this type of approach) that should be addressed:

o The conditional independence assumption (referred by the authors as "CIA") requires that no variables other than those included in their model influence the treatment and the potential outcomes. For analyses that study macroeconomic effects (even though the units of observation are ADM2 regions), this assumption is most likely never fulfilled. The authors argue that it is plausible to assume that the CIA is fulfilled in their setting, because they add a large number of covariates. While I think that the probability for selection on unobserved factors may potentially be low in the authors' setting, this point requires more discussion. The paper motivates the study by citing the "credibility revolution" in economics and criticizes prior methods that aim to tackle unobserved omitted factors in section 2. I think that, particularly when making such strong statements, it is unsatisfactory to say that the problem can be solved by simply adding more variables (although in a quite different methodological setting, this was the core idea underlying previous methodological innovations, e.g. Extreme Bound Analyses in the 1980s/1990s and LASSO regressions in the 2010s). More generally, I think that the empirical analysis would be more convincing if the authors included a brief discussion on how they expect unobserved variables to influence their results.

Thank you for this comment. We have now added a clarifying paragraph to the relevant part in section 2.3 where we openly discuss the problem of omitted variable bias as well as the potential direction of that bias in our setting.

o Another important assumption of the analysis is that the availability of resources is exogenous. On Page 8, the authors write that "along with the recent subnational literature, we argue that the availability of natural resources at the district level is mainly shaped by geology and, therefore, exogenous". Can we really say that this is the case? What about political factors or effort invested to find natural resources? Even geology itself may be endogenous, i.e. when factors rooted in years before 1996 (the starting point of the author's sample) have changed the geological circumstances and facilitated access to natural resources? These factors may well be corelated with growth (via budget constraints) and conflict (by decreasing the potential to be productive in searching for natural resources). But if availability of natural resources would be truly exogenous, then untreated units would never have the chance to be treated at all. If this is case, then I wonder whether they can serve as a plausible control group, i.e. we cannot produce a counterfactual of the treated units using observable characteristics, because the characteristics of treated and untreated units are different "by nature". This problem is not specific to the authors' setting and there is some discussion on this point on Page 9. This discussion, however, is a bit vague and it is not clear to me how severely the "substantial overlap problems" the authors find affect the empirical results.

We think that our discussion of these issues in Section 3.2 is very honest and transparent, especially when compared to most of the previous literature. However, we have added the following qualification to the sentence you have highlight above: "This argument is certainly true for the actual presence of natural resources. However, it is important to acknowledge that it may be endogenous whether these resources are discovered and exploited (e.g., Cust and Harding, 2020)." The overlap problem is probably unrelated to this discussion and remains speculation how relevant it is. However, in the text (middle of section 2.3) it is clearly explained how the necessary extrapolation is performed by the modified causal forest.

o When describing their method, the authors acknowledge that their strategy comes at "the cost of not being able to include that many fixed effects" (Page 6). To account for heterogeneity across units and years, the authors include some "indicator variables", but from their very brief description, it was not clear to me what these variables measure. If the model includes indicator variables for country-years, then how can additional variables that only vary at the country-year level (such as country-level ethnic diversity) add any additional information? The answer may potentially lie in the nature of the indicator variable, but this should be explained briefly in the text.

We now point the reader to the detailed description of the variables included in Section 3.4

o I missed some discussion of the numerical meaning of the results. On Page 15, for example, the authors state that "the estimated effects imply that mining activities increase nighttime lights by 16.0 percent if mineral prices are low, and 48.3 percent if mineral prices are high". This and other similar statements are a bit vague, because nighttime lights are measured on a very counterintuitive scale. Also, the paper is mute about the distribution of nighttime lights. So how large, in economic terms, is an increase in nighttime lights by 16% given the sample upon which these estimates are obtained? I think it is essentially to communicate the meaning of the results in a more reader-friendly way, and the numerical meaning of the estimated effects should be provided directly in the introduction (see also my comments on the exposition of the manuscript).

In the second paragraph of Section 4.1, we now "translate" these effects on nighttime lights into effects on local GDP. In addition, we now also state these effect sizes in the Introduction.

- *In how far are the results affected by units where nighttime lights are top-coded?*

Top-coding is not an issue in our setting. There is only one ADM2 region in our sample where lights are top-coded for the duration of 6 years.

- *The exposition of the manuscript: The paper is very well written and (in most parts) crystal clear about what it does. However, I fear that some parts of the paper are (too) lengthy and sometimes repetitive. Also, some parts of the paper read more like a book than a research paper, particularly at places in the text where the authors refer to interesting but not necessarily important side issues (mainly in the introduction and the description of the method). While I enjoyed reading the text and while there is clearly personal taste in writing, I think that the communication of the results could be strengthened by streamlining the text a bit, particularly in the introduction. Some specific examples: It takes the reader until Page 3 to learn what the paper does and until Page 4 to learn about the main results. Before, there are interesting but lengthy discussions about oil production in Guyana and the credibility revolution in economics. These are clearly interesting topics, but their relevance for the paper is minor compared to the papers' strategy and main results.*

We have streamlined the early parts of the introduction. Thereby we have cut the example of Guyana as well as the statement on the credibility revolution. As a result, we now start discussing our own paper in the top-half of page 2.

In section 2.2, we cut some sentences that are not strictly necessary to understand the estimators. However, as we assume that most of the readers are not familiar with causal forests, we keep most of the rather extensive description of this estimation method.

Reviewer #2:

I am glad for the opportunity to read and review the manuscript "Institutions and the Resource Curse: New Insights from Causal Machine Learning". In general, the article attempts to bring a contribution to the literature on the nature of the resource curse from an institutional perspective, by using causal machine learning tools.

In my opinion, the article is very interesting and valuable given the topic and methodology. It may be suitable for publication in PLOS ONE upon a minor revision. I list my concerns regarding the manuscript below (in the order in which they appear in the manuscript):

1. The part of the Introduction on the character of causal machine learning (p. 3) can be restructured as it contains several repetitions in the current form

We have now rewritten the relevant parts of the introduction and ensured that there are no more repetitions.

2. The non-parametric/non-linear estimation strategy is well-justified; the description of the modified causal forest estimator is transparent as well as the relevance of ATEs, GATEs and IATEs

Thank you.

3. The authors can be straightforward about the advantages of using light intensity as a proxy for economic development – especially the availability of data at the subnational level; on the other hand, potential caveats of this approach should be described as well

We have now included a discussion of the recent literature highlighting the shortcomings of the use of nighttime light data as a proxy for subnational economic activity (e.g. Chen and Nordhaus 2015; Gibson et al. 2021; Asher et al. 2021) and discussed the potential implications for our study at the end of the relevant paragraph in section 3.1.

4. It can be stated that local conflicts may be of various types, intensity and duration, so the disadvantages of using a binary variable in this context should be provided

We have now included a justification for using a binary outcome variable as well as the drawbacks of this decision at the end of the relevant paragraph in section 3.1.

5. More arguments for choosing the particular geographical and time scope are suggested to be provided.

We have added additional arguments for our choice of geographical resolution and the sample period at the beginning of section 3.

6. The main text should include at least a brief review of the confounding variables, as well as descriptive stats analysis (also to understand better how these factors are included in the models)

We now present a short description of each confounding variable in the main text and moved the descriptive statistics table to Section 3.4. We have left the detailed explanation on how the different variables were calculated as well as the underlying sources in Appendix 2.

7. Given that the authors aim to address the role of institutions in the context of the resource curse, they may consider supplementing the article with XAI tools (e.g. Shapley values or feature importance index) to see the contribution of institutions (constraints on the executive/quality of government) to the model

Thank you for the suggestion. We have now included a new Table 4 that, for the case of NTL, presents the variable importance statistics in terms of %-lost of base value for the most important variables.

8. It would be also beneficial to relate the obtained results to the existing literature in a broader manner

We discuss the relation of our results to those in the previous literature at the end of the first and the third paragraphs of Section 4.1.

9. Proofreading of the manuscript is recommended as it contains some typos, punctuation mistakes and minor stylistic errors

We have sent the revised manuscript to a professional proofreading service.

I hope these comments can be useful in improving the paper.

Reviewer #3:

Short summary:

Using district-level panel data from Africa and applying a causal Machine Learning approach, the paper provides novel insights on the effects of natural resouces on economic development and conflict. Thereby, special attention is paid on the question whether these effects depend on the level of institutional quality. The main results are that well-functioning institutions increase the positive effects of mining activities on development and reduces their negative effect on conflict. By contrast, institutional quality plays no role for the effects of mineral prices on these outcomes.

General assessment.

From my perspective, the paper is a valuable contribution to the literature. I also believe that the papers fits to the journal. I have two major comments and some smaller points. You can find them below.

Major comments.

(1) My first set of major comments are related to your measures of institutional quality.

(i) You use the Quality of Government indicator from the ICRG rating by the PRS Group to measure economic institutional quality. I think that this measure is a bad choice for several reasons. First, the way of how the PRS Group produces their measures is a black box as their are no coding rules available. Furthermore, the measure is based on various arbitrary decisions: for instance, there is no theoretical explanation for why "corruption" as well as "law and order" are measured with 0-6 scale, while a 0-4 scale is used to measure bureaucratic efficiency. Another problem is the aggregation procedure that is used to combine the three components. More specificaay, a recent study in the European Economic Review suggests that indices that are based on additive aggregation methods are likely to produce upward-biased estimates (see Gründler and Krieger, 2022).

From my perspective, the popularity of the measures developed by the PRS Group decreased quite a lot over the last years. I even believe that using this data can be regarded as outdated. My suggestions is thus to use another data source. The V-DEM is probably the best choice for your purpose.

We now acknowledge at the beginning of Section 3.3 that there exists a vast set of indicators of institutional quality, which often cover related but not identical aspects of institutional quality, or which measure the same aspect based on different methodological approaches. There we also acknowledge that it is in no way obvious which measures are best suited and we clarify what selection criteria have guided our choice.

The aim of this paper is to apply a new estimator to reassess the relationship between institutions and the effects of resource rents and compare our results to those of existing studies. The original papers that we use as a benchmark, such as Mehlum et al. (2006a, 2006b) used the

Quality of Government indicator from the ICRG rating by the PRS Group and we use the same data for reasons of consistency. As we aim to investigate what new insights we can gain from using a novel estimator – the modified causal forest estimator –, we deliberately use variables that have already been used in the previous literature. Otherwise, it would be difficult to know whether different results are due to the use of different variables or the use of the novel estimator. More specifically, we find that the argument by Mehlum et al. (2006a, 2006b) holds for new mining activities, but not for higher resource prices. Given that we use the ICRG Quality of Government indicator we can be sure that this novel finding is not driven by the use of a different indicator of institutional quality. We make this argument in general terms at the beginning of Section 3 right before Section 3.1, and also at the beginning of Section 3.3 when introducing the ICRG Quality of Government indicator.

Finally, we are not sure whether the concern of upward bias is warranted in our case given the differences between the exercise conducted by Gründler and Krieger (2022) and by us. Gründler and Krieger (2022) present linear OLS and IV estimates of the direct effect of political institutions on economic growth, while we use a non-linear estimator and mainly use the indicators of institutional quality to compute the group-average treatment effects (GATEs) when trying to find out how the effect of resource rents on economic development or conflict varies depending on institutional quality. Thereby, we stratify the ICRG Quality of Government indicator into a number of groups and do not use the exact score. Obviously, the aggregation procedure used in the computation of this (or any other) indicator may still impact our result, but it is a priori unclear whether it would lead to an upward or a downward bias. We now however cite Gründler and Krieger (2022) in footnote 17 where we illustrate the vast numbers of indicators of institutional quality and the general type of differences between them.

(ii) You use a sub-component of the POLITY index to measure political institutional quality. I know that POLITY is widely used, but most people who extensively thought about the measurement of political institution heavily criticize this measure (see e.g.\ Munck and Verkuilen (2002, Comparative Political Studies), Cheibub et al. (2010, Public Choice), Boese (2019, International Area Studies Review), Gründler and Krieger (2021, European Journal of Political Economy)). Put differently, I think that you should dig deeper into the literature on the measurement of democracy in order to find better alternative.

We now argue in the corresponding paragraph in Section 3.3 that the use of this measure is consistent with our selection criteria mentioned at the beginning of Section 3.3: There are certainly no indicators of political institutions that have been used more often than those from the Polity IV project, and we think that the commonly used Constraints on the Executive indicator captures the notion of political institutions that constrain the executive. We also include a new footnote (19) where we refer the reader to the references provided by the referee.

(iii) From a more conceptual perspective, I wonder why you consider the quality of economic and political institutions separately from each other in your analysis. There is large number of studies, suggesting that economic institutional quality is influenced by the type of political regime.

We agree in principle. However, we really wanted to keep the ICRG Quality of Government indicator used by Mehlum et al (2006a, 2006b), which we see as an indicator of the quality of

the economic institutions. We now mention in footnote 18 in Section 3.3 that economic institutions may be endogenous to political institutions. Also, note that we illustrate the difference between our two indicators in footnote 20 at the end of Section 3.3.

(iv) Both in the introduction and the conclusion, you stress the policy relevance of your study. In principle, I agree on this point, but I think the paper improves if you disentangle the role of the different aspects of institutional quality. For instance, from a policy maker perspective, it would be nice to know whether fighting off corruption is worse or better than improving the efficiency of the bureaucracy or the independece of the courts. The V-DEM dataset might be helpful here as well.

We agree that this is question is both academically interesting and important for policy advise. However, this paper has a different objective, namely to generate new insights by investigating the validity of a well-established argument using a novel estimator. Therefore, we don't explore this question in this paper, but recommend it for future research in the last paragraph of the Conclusions.

(2) In your empirical analysis, you try to control for a large number of potential confounders. I wonder a bit about the choice. More specifically, you add a large number of additional measures of institutional quality. I am quite concerned about this approach as some of them even measure the same the variables that you use to study effect heterogeneity. You also add the Polity index as control variable, which is (by construction) depending on the measure of executive constraints. For me, it is not clear why you do this and I wonder whether these additional measures of institutional quality are bad controls and thus bias your main estimates.

As highlighted in response to your comment 1, there exist many measures of institutional quality that aim to capture related concepts and often do so applying different methodological approaches. Given that we are not interested in the direct effect of institutional quality per se, but in the effect of natural resource rents and in how this latter effect differs depending on the popular measures of institutional quality, we believe that it makes sense to control for the direct effect of various aspects of institutional quality by controlling for more than just these two measures. In short, we do not see why they should be bad controls considering our research question.

Other comments.

(3) You do not find that institutional quality influences the effects on mineral prices on economic development and conflict. I would like to see some words on why this might be the case, especially as your result is at odds with the papers by Mehlum et al.

We discuss a possible reason for why some of our results are at odds with the general argument based on Mehlum et al. (2006a, 2006b) in a new paragraph at the very end of Section 4.2. We thereby build on the theoretical model by Mehlum et al. (2006a).

(4) I wonder whether you should exclude districts from the control group, as there are some studies that find spillover effects.

The evidence on spillover effects of natural resource rents on economic development or conflict in neighbouring areas is inconclusive. For example, Berman et al. (2017, Table 2, column (3)) find some limited spatial conflict spillovers, but these spillovers are rather small despite the use of relatively small grid cells. Mamo et al. (2019, p. 28) find "little evidence for significant [economic] spillovers to other districts," as they state in the abstract. Amarasinghe et al. (2020a) find some economic spillovers if districts are well connected by roads or part of the same ethnic homeland.

However, importantly, none of these papers exclude some districts or grid cells from the analysis. Therefore, to ensure comparability to the previous literature, we also keep all districts in our analysis. Notice that if there were significant spillovers, then the assignment of non-mining districts contiguous to mining districts would lead to attenuation bias. We now discuss this possibility in footnote 16 in Section 3.2.

(5) On page 3, you write: "We argue that our non-parametric approach is preferable or, at least, a worthwhile alternative, because linear fixed-effects specifications are based on a set of assumptions that may not hold." Of course, this is true, but your approach is also based on assumptions that might be violated. I think you rather write that your approach relaxes some of the assumptions of the linear fixed-effect approach.

We now tuned down the advantages of our approach and emphasise that it is a worthwhile alternative as it does not depend on these assumptions. However, we do not claim superiority.

(6) For my taste, Section 2 is a bit too long.

We changed this section to the extent that there is now a somewhat deeper discussion of the relation to the recent developments in flexible difference-in-difference estimation (section 2.1) and we cut a few sentences in section 2.2. However, we also figured that many readers might have a limited knowledge about the new causal machine literature and will thus value detailed, nontechnical explanations. We also decided that the identification part is too important for judging the credibility of our results to be cut.

(7) In footnote 12, you write that you add 0.01 to the measure of nightlight intensity before taking the log. Given the recent working paper by Chen & Roth ("Log-like? Identified ATEs defined with zero-valued outcomes are (arbitrarily) scale-dependent"), I wonder whether this approach creates biases.

This is indeed an interesting paper. To ensure comparability to the previous literature, e.g., the closely related paper by Mamo et al. (2019) as well as many other papers, we keep this transformation but acknowledge the work of Chen and Roth (2023) in the footnote 13 in Section 3.1.

New References:

Asher, S., Lunt, T., Matsuura, R. and P. Novosad (2021): Development research at high geographic resolution: an analysis of night lights, firms, and poverty in India using the SHRUG open data platform, *World Bank Economic Review*, 35(4), 845–871.

Boese, V. A. (2019). How (not) to measure democracy. *International Area Studies Review*, 22(2), 95–127.

Cheibub, J.A., J. Gandhi, and J.R. Vreeland (2010): Democracy and dictatorship revisited. *Public Choice*, 143, 67–101.

Chen, J., and J. Roth (2023): Log-like? Identified ATEs defined with zero-valued outcomes are (arbitrarily) scale-dependent, arXiv:2212.06080.

Chen, X. and W.D. Nordhaus (2011): Using luminosity data as a proxy for economic statistics, *Proceedings of the National Academy of Sciences*, 108(21), 8589–8594.

Chen, X. and W.D. Nordhaus (2015): A test of the new VIIRS lights data set: Population and economic output in Africa, *Remote Sensing*, 7(4), 4937-4947.

Cust, J., and T. Harding (2020): Institutions and the location of oil exploration, *Journal of the European Economic Association*, 18(3), 1321-1350.

Gibson, J., Olivia, S., Boe-Gibson, G., and C. Li (2021): Which night lights data should we use in economics, and where? *Journal of Development Economics*, 149, 102602.

Goertz, G. (2020): *Social Science Concepts and Measurement: New and Completely Revised Edition*. Princeton University Press.

Gründler, K., and T. Krieger (2021): Using Machine Learning for measuring democracy: A practitioners guide and a new updated dataset for 186 countries from 1919 to 2019. *European Journal of Political Economy*, 70.

Gründler, K., and T. Krieger (2022): Should we care (more) about data aggregation? *European Economic Review*, 142(1), 104010.

Munck, G. L., and J. Verkuilen, (2002). Conceptualizing and Measuring Democracy: Evaluating Alternative Indices. *Comparative Political Studies*, 35(1), 5–34.
